# 超高效液相色谱-串联质谱法测定人尿及灰尘中*N*-（1，3-二甲基丁基）-*N*′-苯基-对苯二胺-醌

**DOI:** 10.3724/SP.J.1123.2025.02010

**Published:** 2025-09-08

**Authors:** Zhuliangzi LU, Fenfang DENG, Zhijun BAI, Rongfei PENG, Lei TAN

**Affiliations:** 广州市疾病预防控制中心，广州医科大学公共卫生研究院，广东 广州 510440; Guangzhou Center for Disease Control and Prevention，Institute of Public Health，Guangzhou Medical University，Guangzhou 510440，China

**Keywords:** 超高效液相色谱-串联质谱法, *N*-（1，3-二甲基丁基）-*N′*-苯基-对苯二胺-醌, 尿液, 灰尘, ultra performance liquid chromatography-tandem mass spectrometry （UPLC-MS/MS）, *N*-（1，3-dimethylbutyl）-*N′*-phenyl-*p*-phenylenediamine-quinone （6PPD-Q）, urine, dust

## Abstract

为有效监测*N*-（1，3-二甲基丁基）-*N′*-苯基-对苯二胺-醌（6PPD-Q）在人群中内外暴露水平，建立了人体尿液及灰尘中6PPD-Q的超高效液相色谱-串联质谱（UPLC-MS/MS）分析方法。通过系统优化前处理过程及色谱、质谱分析条件，确定了尿液及灰尘样品中6PPD-Q的最佳检测条件。具体方法如下：尿液样本中加入0.1 ng内标^13^C_6_-6PPD-Q平衡30 min后，加入谷胱甘肽和NaCl，经乙酸乙酯超声萃取两次；灰尘样本中加入1.0 ng内标^13^C_6_-6PPD-Q，经正己烷超声萃取两次。所得萃取溶剂合并，浓缩至近干，复溶后用于进样分析。采用含0.01%甲酸的10 mmol/L乙酸铵溶液和乙腈作为流动相进行梯度洗脱，Phenomenex Kinetex F5（100 mm×3 mm， 2.6 μm）作为色谱分析柱，在正离子电喷雾（ESI^+^）多反应监测（MRM）模式下检测，内标法定量。在优化的实验条件下，6PPD-Q在0.01~4.00 μg/L（尿液）和0.01~20.0 μg/L（灰尘）范围内具有良好的线性，相关系数分别为0.999 9和0.999 3，检出限分别为0.6 ng/L（尿液）和0.018 ng/g（灰尘）。在低、中、高3个加标水平下，6PPD-Q的加标回收率为90.3%~94.1%，日内精密度为0.9%~5.9%，日间精密度为1.1%~6.3%。基质效应评估结果表明，采用同位素标记内标校正法可有效减少尿液及灰尘基质中6PPD-Q定量分析时的基质干扰。将该方法应用于120份人群尿液样本的分析，尿液样本中6PPD-Q的检出率为74.2%，检出范围为<LOD~13 ng/L，平均值为2 ng/L，中位值为1 ng/L。31份室内灰尘样本中6PPD-Q均有检出，检出范围为1.8~24.9 ng/g，平均含量为5.23 ng/g，中位含量为3.05 ng/g。本方法准确、可靠，且灵敏度高，适用于人体尿液及灰尘中6PPD-Q的检测。

## Abstract (en)

An ultra performance liquid chromatography-tandem mass spectrometry （UPLC-MS/MS） method was established to determine *N*-（1，3-dimethylbutyl）-*N′*-phenyl-*p*-phenylenediamine-quinone （6PPD-Q） in human urine and dust in order to understand the internal and external exposure levels in humans. The sample preparation conditions were systematically investigated and the chromatographic conditions and MS parameters were optimized. Briefly， internal standard ^13^C_6_-6PPD-Q （0.1 ng） was added to a urine sample. Glutathione and NaCl were added after equilibration for 30 min. The mixture was then twice ultrasonically extracted with ethyl acetate. Internal standard ^13^C_6_-6PPD-Q （1.0 ng） was also added to a dust sample and the mixture was twice ultrasonically extracted with *n*-hexane. The combined organic phases were concentrated to near-dryness and then redissolved for instrumental determination， which was performed on a Phenomenex Kinetex F5 column （100 mm×3 mm， 2.6 μm）， with the target analyte gradient eluted using 10 mmol/L ammonium acetate aqueous solution containing 0.01% formic acid and acetonitrile. Positive electrospray ionization （ESI^+^） and multiple reaction monitoring （MRM） modes were used for identification purposes， and an isotope-labeled internal standard was added for quantification. Good linearities were achieved under the optimized conditions within the 0.01–4.00 and 0.01–20.0 μg/L ranges for urine and dust， respectively， with correlation coefficients of 0.999 9 and 0.999 3， respectively. Limits of detection （LODs） were 0.6 ng/L （urine） and 0.018 ng/g （dust）. Spiked recoveries of 6PPD-Q were 90.3%–94.1% at low， medium， and high spiked levels， with intra-day and inter-day precisions of 0.9%–5.9% and 1.1%–6.3%， respectively. Matrix-effect investigations revealed that 6PPD-Q exhibited weak matrix effects in urine and dust after correction with the isotopic internal standard. The developed method was used to analyze 120 human urine samples， which led to a 6PPD-Q detection frequency of 74.2%， with mass concentrations ranging from <LOD to 13 ng/L， with average and median mass concentrations of 2 and 1 ng/L， respectively. However， 6PPD-Q was detected with a frequency of 100% in 31 indoor dust samples with contents of 1.8–24.9 ng/g， and average and median contents of 5.23 –3.05 ng/g， respectively. The developed method is accurate， reliable， highly sensitive， and it is suitable for the rapid determination of 6PPD-Q in human urine and dust samples.

*N-*（1，3-二甲基丁基）-*N′*-苯基-对苯二胺-醌（6PPD-Q）是橡胶轮胎抗氧化剂*N*-（1，3-二甲基丁基）-*N′*-苯基-对苯二胺（6PPD）释放至环境后形成的氧化产物^［1，2］^。已有研究表明，6PPD-Q在环境的浓度与6PPD相当甚至更高，并且对水生生物的毒性更强，能够危害水体中的多种鱼类^［3-5］^。除了鱼类，6PPD-Q还会导致小鼠的肝、肾、肺、脾、睾丸和脑受损以及巨噬细胞和白细胞介素-6、白细胞介素-22水平升高^［6，7］^。Wu等^［8］^研究发现，6PPD-Q可在哺乳动物细胞和水生生物中形成DNA加合物。尽管6PPD-Q的水生毒性得到广泛关注，但由于缺乏这种化学物质对不同器官潜在影响的研究，其对哺乳动物和人类的不良影响以及毒性机制仍不清楚^［9］^。6PPD-Q在多个国家的空气、灰尘、土壤、污水等环境介质中均有检出。已有研究^［10，11］^报道了在电子垃圾回收车间灰尘、室外灰尘、居民公寓室内灰尘等样品中检测到6PPD和6PPD-Q，6PPD的中位含量分别为4.01、3.92、10 ng/g，6PPD-Q的中位含量分别为36.4、10.2、9.5 ng/g。Seiwert等^［12］^在德国污水处理厂的进出水中均检测到了6PPD-Q，平均值分别为0.105 μg/L和0.052 μg/L。6PPD-Q的来源途径及存在介质较广，会通过呼吸、皮肤接触和摄食等途径进入人体，并产生健康危害。因此可以通过测定人体生物样本及灰尘中6PPD-Q水平来了解人群的内外暴露水平及变化规律，为6PPD-Q的污染防治以及人群健康风险评价提供科学依据。

目前，6PPD-Q的检测方法主要包括高效液相色谱法（HPLC）^［12，13］^、超高效液相色谱-串联质谱法（UPLC-MS/MS）^［14-16］^、超高效液相色谱-高分辨质谱法（UPLC-HRMS）^［17，18］^。Hiki等^［14］^通过UPLC-MS/MS测定了主干道与住宅道路灰尘中6PPD-Q的含量。Ji等^［19］^建立了改进的QuEChERS结合高效液相色谱-串联质谱法检测鱼类和蜂蜜中6PPD和6PPD-Q的方法，方法定量限为0.000 43~0.001 mg/kg，回收率为73.3%~108.3%。但是目前有关6PPD-Q的检测样本多为水样、灰尘或者食品等，哺乳动物及人体生物样本中6PPD-Q的研究较少^［20］^。Zhang等^［21］^建立了UPLC-HRMS测定小鼠粪便及尿液样本中的6PPD-Q。Liang等^［22］^采用UPLC-MS/MS测定了华南地区一般城市人口母乳样本中的6PPD和6PPD-Q，方法定量限分别为4.03 pg/mL和4.89 pg/mL，但仅在52%的样本中检测到了6PPD，而未检测到6PPD-Q。Liu等^［23］^采用UPLC-MS/MS对100名志愿者的血浆和尿液样本中的6PPD-Q进行了检测，方法定量限分别为0.04 ng/mL（血浆）和0.05 ng/mL（尿液）。由于人体尿液可以无损采集，且收集相对容易，尿液样本已被广泛用于评估人类环境污染物的暴露水平^［24，25］^。因此，为了解6PPD-Q在人群中内外暴露水平，亟需建立一种可靠、灵敏的方法用于检测人体尿液样本及灰尘中的6PPD-Q。近年来，超高效液相色谱-串联质谱技术具有灵敏度高、分析速度快、应用广泛的优点^［26，27］^，尤其针对基质复杂的生物样本，分离效果好，准确度高，已成为环境污染物分析的常用技术，适合各类样品中污染物的定性定量检测。

因此，本研究通过超高效液相色谱-串联质谱法建立了人体尿液和室内灰尘中6PPD-Q的检测方法，对前处理条件进行了筛选优化，并应用于健康人群6PPD-Q内外暴露的监测，可以为后续人群6PPD-Q内外暴露水平监测及健康风险评估提供数据积累和技术支撑。

## 1 实验部分

### 1.1 仪器、试剂与材料

Acquity H-Class Plus UPLC/Xevo TQ-XS超高效液相色谱-串联质谱仪（美国Waters公司），FV64型氮吹仪（广州得泰仪器科技有限公司），2-16型台式高速离心机（美国Sigma公司），涡流混匀器（美国Scientific公司），Milli-Q超纯水机（德国Merck公司）。

6PPD-Q（100 mg/L，以甲醇为溶剂）、^13^C_6_-6PPD-Q（10 mg/L，以甲醇为溶剂）均购于天津阿尔塔科技有限公司。甲酸（LC-MS级）、谷胱甘肽（分析纯）、氯化钠（分析纯）均购于上海阿拉丁生化科技股份有限公司，乙酸铵（美国SCIEX公司），二氯甲烷（DCM）、正己烷（HEX）、乙酸乙酯（EA）、乙腈（ACN）、甲醇均为LC-MS级，购于德国Merck公司，实验用水均来自Milli-Q超纯水机所制得的超纯水。

### 1.2 溶液的配制

6PPD-Q标准使用液：准确移取1.0 mL的100 mg/L 6PPD-Q标准溶液于10 mL容量瓶中，用乙腈定容为10 mg/L的6PPD-Q标准中间液，再用乙腈逐级稀释为100 μg/L的6PPD-Q标准使用液，于-20 ℃下冻存备用。

内标^13^C_6_-6PPD-Q使用液：准确移取1.0 mL的10 mg/L ^13^C_6_-6PPD-Q标准溶液，用乙腈定容为1.00 mg/L的^13^C_6_-6PPD-Q内标中间液，再用乙腈逐级稀释为5.00 μg/L的^13^C_6_-6PPD-Q内标使用液，于-20 ℃下冻存备用。

尿液系列标准溶液的配制：以50%（v/v）乙腈水溶液为溶剂将6PPD-Q标准使用液配制成质量浓度为0.01、0.02、0.05、0.10、0.50、1.00、2.00、4.00 μg/L的系列标准溶液。在其中加入^13^C_6_-6PPD-Q内标使用液，使系列标准溶液含内标质量浓度为0.50 μg/L。

灰尘系列标准溶液的配制：以甲醇为溶剂将6PPD-Q标准使用液配制成质量浓度为0.01、0.02、0.05、0.10、0.50、1.00、2.00、4.00、10.0、20.0 μg/L的系列标准溶液。在其中加入^13^C_6_-6PPD-Q内标使用液，使系列标准溶液含内标质量浓度为2.00 μg/L。

### 1.3 样品采集和前处理

本研究包含120份广州市健康人群的尿液样本，项目获广州市疾病预防控制中心伦理委员会审查批准（批准号：GZCDC-ECHR-2020P0011）。

人体尿液：收集中段晨尿于洁净的离心管中，于-80 ℃避光冷冻保存。冷冻的尿液样品从-80 ℃冰箱取出后，于4 ℃冰箱中解冻12 h，再取出恢复至室温。将恢复至室温的尿样涡旋混匀，准确移取1 mL尿样于1.5 mL离心管中，加入0.1 ng ^13^C_6_-6PPD-Q的内标（20 μL 5.00 μg/L的^13^C_6_-6PPD-Q内标使用液），平衡30 min后加入0.2 mL 1 mmol/L谷胱甘肽溶液（抑制样本中6PPD的潜在氧化转化），加入0.4 g NaCl后，加入3 mL乙酸乙酯，涡旋振荡10 min，超声提取10 min。以5 000 r/min离心5 min后，取上层有机相转移到另一个干净的离心管中。尿样再用相同操作步骤，重复提取一次。将两次提取的萃取液合并，在压力为41.3 kPa的氮气、30 ℃的水浴条件下浓缩至近干，再用200 μL 20%（v/v）乙腈水溶液复溶，用于仪器分析。

灰尘：31份室内灰尘样本从广州市31个不同的居民住宅收集。用甲醇预清洁过的刷子和铲子采集客厅和卧室内桌面和地面沉降的灰尘样本，收集于干净的铝箔上包裹好，放入自封袋内。将采集的灰尘样本风干、均质、筛分（60目），用干净的铝箔包裹好，密封在棕色玻璃容器中，储存在-20 ℃，用于后续分析。准确称取0.05 g灰尘样品，加入1.0 ng ^13^C_6_-6PPD-Q的内标（200 μL 5.00 μg/L的^13^C_6_-6PPD-Q内标使用液），加入3 mL正己烷，超声提取20 min，4 000 r/min转速下离心10 min。再用相同操作步骤，重复提取一次。将两次提取的萃取液合并，在压力为41.3 kPa的氮气、30 ℃的水浴条件下浓缩至近干，再用500 μL 20%（v/v）乙腈水溶液复溶，用于仪器分析。

### 1.4 仪器条件

Kinetex F5色谱柱（100 mm×3 mm，2.6 μm，美国Phenomenex公司），隔离柱（50 mm×2.1 mm，美国Waters公司），柱温为40 ℃。流动相为含0.01%甲酸的10 mmol/L乙酸铵溶液和乙腈，梯度洗脱程序如表1所示。进样体积为3.0 μL。

**表 1:** 梯度洗脱程序

Time/min	Flow rate/（mL/min）	*φ*（A）/%	*φ*（B）/%
Initial	0.3	90	10
1.5	0.3	90	10
4.0	0.3	0	100
11.0	0.3	0	100
12.0	0.3	90	10
15.0	0.3	90	10

A： 10 mmol/L ammonium acetate aqueous solution （containing 0.01% formic acid）； B： acetonitrile.

离子源为电喷雾离子源（ESI），扫描模式为正离子模式，检测方式为多反应监测（MRM）。离子源温度：150 ℃；脱溶剂温度：500 ℃。毛细管电压：0.80 kV。去溶剂气流量：800 L/h；锥孔气流量：150 L/h；碰撞气流量：0.15 mL/min。其他质谱参数见表2。

**表 2:** 6PPD-Q及内标的质谱参数

Compound	Retention time/min	Parent ion （*m*/*z*）	Daughter ions （*m*/*z*）	Cone voltage/V	Collision energies/eV
6PPD-Q	5.13	299.1	241.1^*^， 215.1	60	40， 24
^13^C_6_-6PPD-Q	5.13	305.2	247.1^*^， 221.1	60	40， 24

^*^ Quantitative ion.

## 2 结果与讨论

### 2.1 色谱条件优化

6PPD-Q不易溶于水，可以通过离子作用和疏水相互作用吸附在不同材料表面。色谱柱类型、流动相组成等都会影响6PPD-Q的保留行为和色谱峰形。本研究考察了4种不同色谱柱（Kinetex F5（100 mm×3 mm，2.6 μm）、Acquity CSH fluoro-phenyl（100 mm×2.1 mm，1.7 μm）、Atlantis T3（50 mm×2.1 mm，3 μm）、Acquity BEH C_18_（50 mm×2.1 mm，1.7 μm））的分离效果。结果如图1所示，流动相为乙腈-水体系时，6PPD-Q经4种不同的色谱柱流出，化合物峰形均较好，但信号峰高差异较大，其中F5柱的保留能力最强，质谱响应信号最高。6PPD-Q经不同色谱柱流出后信号峰高的差异可能是不同色谱柱的固定相类型、键合方式和表面修饰影响目标物与固定相的相互作用，这些差异会导致峰形、峰宽和保留行为的变化，从而导致6PPD-Q信号峰高有所差异。另外，不同色谱柱可能需要优化流动相组成和流速才能呈现最佳性能，色谱柱比如孔径和粒径不同会导致系统死体积等不同，从而影响峰扩散，这些因素也会影响最终的峰形和峰高。而实验测试所用的色谱柱都不是全新的色谱柱，较老或使用频繁的色谱柱可能表现出效率降低和峰形变差等情况。综上，选择F5柱作为色谱柱进行后续分析实验。

**图1:**
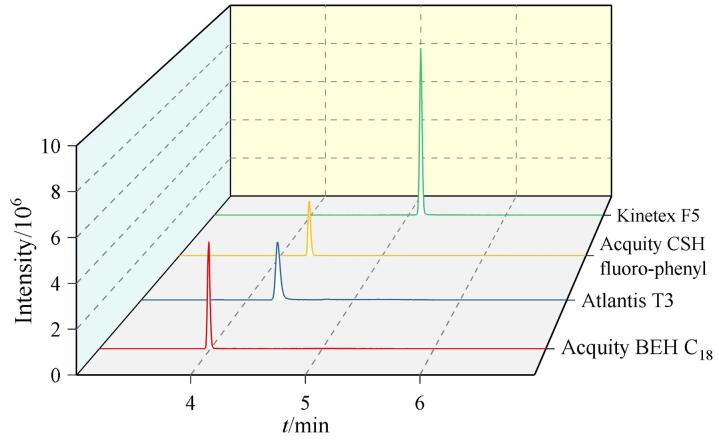
使用不同色谱柱时6PPD-Q （1.0 μg/L）的色谱图

在此基础上，实验还考察了不同流动相对6PPD-Q色谱峰形和灵敏度的影响。以乙腈作为有机相，水相选择不同浓度的乙酸铵溶液（2、5、10 mmol/L）和不同体积分数（0.01%、0.05%、0.1%、0.2%）的甲酸水溶液分别进行实验。发现不同浓度的乙酸铵溶液和不同体积分数的甲酸水溶液作为水相时，6PPD-Q的峰形差别不大。其中10 mmol/L乙酸铵溶液和0.01%甲酸水溶液作为水相时，质谱响应信号最高。进一步考察了含有0.01%甲酸的10 mmol/L乙酸铵溶液作为水相，发现6PPD-Q色谱峰的响应信号是10 mmol/L乙酸铵溶液作为水相时的1.1倍。因此，选择含有0.01%甲酸的10 mmol/L乙酸铵溶液作为水相。确定水相后，实验对比了有机相为甲醇或乙腈对6PPD-Q保留能力以及峰形的影响。甲醇对6PPD-Q的保留能力较乙腈稍强，但有机相为甲醇时6PPD-Q响应信号明显低于乙腈。最终选择含有0.01%甲酸的10 mmol/L乙酸铵溶液-乙腈作为流动相。

优化条件下，6PPD-Q及内标标准溶液的提取离子色谱图见图2。

**图2:**
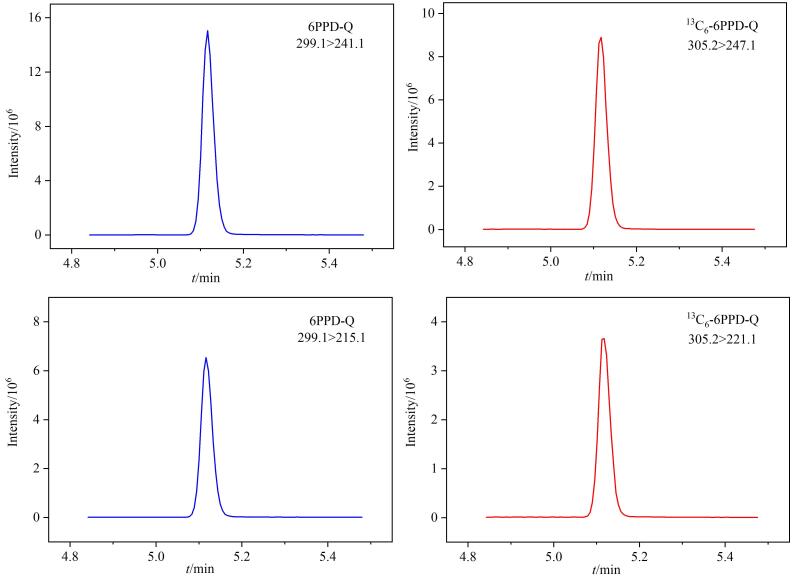
6PPD-Q（2.0 μg/L）及内标（0.5 μg/L）的提取离子色谱图

### 2.2 溶剂空白的考察

因6PPD广泛应用于各种橡胶制品，因此不排除实验采用的各种试剂、耗材中可能检出其氧化产物6PPD-Q，因此，必须对溶剂空白进行严格考察，只有空白中6PPD-Q的浓度低于方法检出限，建立的方法才能提供可靠的数据。在优化的色谱、质谱条件下，依次将溶剂空白（20%乙腈水溶液）、0.05 μg/L 6PPD-Q标准溶液（^13^C_6_-6PPD-Q：0.5 μg/L）注入仪器分析，再将溶剂空白连续进样两次，得到的提取离子色谱图如图3所示。

**图3:**
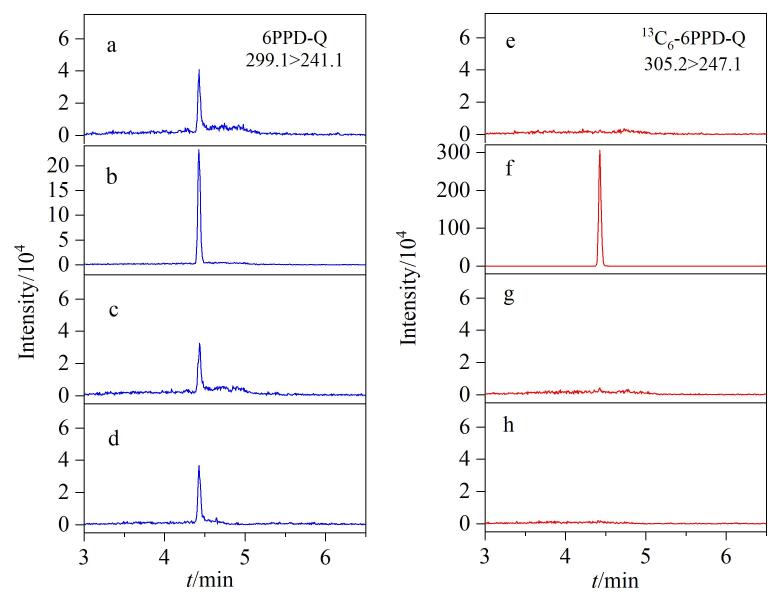
安装隔离柱前连续进样的样品的提取离子色谱图 a， e. solvent blank； b， f. standard solution； c， g. first solvent blank right after standard solution； d， h. second solvent blank right after standard solution.

如图3a所示，安装隔离柱进行隔离之前，溶剂空白在6PPD-Q保留时间处出峰，而在^13^C_6_-6PPD-Q保留时间处未出峰（图3e）。在进样0.05 μg/L 6PPD-Q（^13^C_6_-6PPD-Q：0.5 μg/L）标准溶液（图3b）之后，接着对溶剂空白连续进样两次，其提取离子色谱图如图3c和3d所示，溶剂空白在6PPD-Q保留时间处仍有出峰，且与第一次溶剂空白中6PPD-Q的峰高响应接近，后续一次进样的溶剂空白6PPD-Q仍有残留，且峰高响应变化不大（如图3d）。值得注意的是，在^13^C_6_-6PPD-Q保留时间处连续两个样本均未出峰（如图3g、3h所示）。以上结果表明流动相中可能存在6PPD-Q，需要进行控制，否则将影响方法定量的准确性。因此，我们在仪器液相二元泵高压混合之后与进样系统之间安装了隔离柱，再重复前述实验，得到的提取离子色谱图如图4所示。

**图4:**
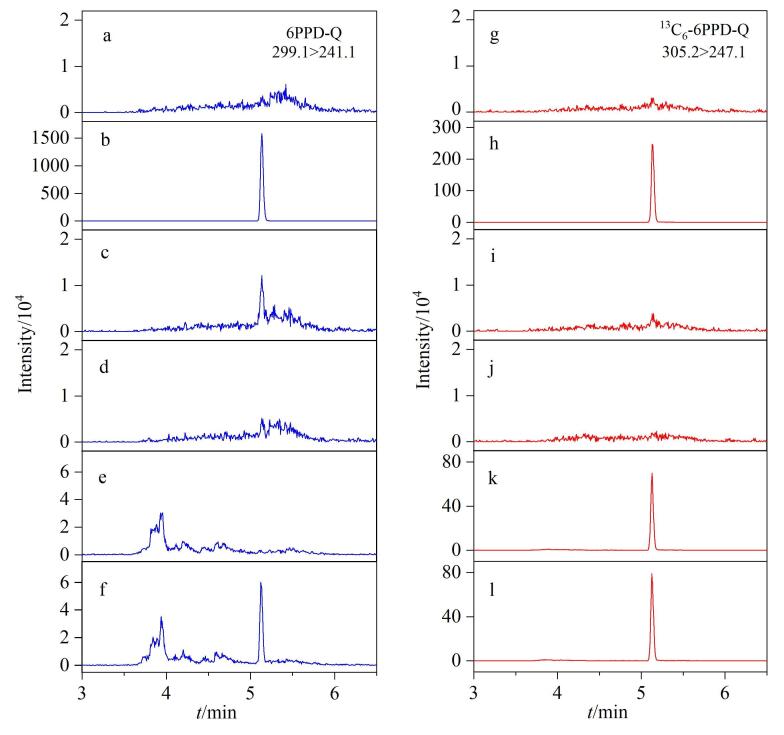
安装隔离柱后连续进样的样品的提取离子色谱图 a， g. solvent blank； b， h. standard solution； c， i. first solvent blank right after standard solution； d， j. second solvent blank right after standard solution； e， k. urine sample； f， l. spiked urine sample.

如图4a所示，安装隔离柱后，溶剂空白中未出现6PPD-Q的定量离子峰。在对4.0 μg/L 6PPD-Q（^13^C_6_-6PPD-Q：0.5 μg/L）的标准溶液（图4b）进样分析之后，继续对溶剂空白进行进样分析，所提取离子色谱图（图4c、4i）显示6PPD-Q和^13^C_6_-6PPD-Q在目标保留时间处均出峰，说明6PPD-Q和^13^C_6_-6PPD-Q有一定的残留，6PPD-Q的残留量约为0.08%（与4.0 μg/L 6PPD-Q的峰面积之比），而继续进样溶剂空白结果如图4d和4j所示，6PPD-Q和^13^C_6_-6PPD-Q的定量离子峰峰高大幅降低，残留量约为0.01%，接近基线。说明在高浓度的标准溶液后接着进样存在一定的柱残留，但通过连续两针空白的清洗基本可以去除。图4e、4k和图4f、4l分别为实际尿样和加标尿样（加入6PPD-Q 0.05 μg/L，^13^C_6_-6PPD-Q 0.5 μg/L）的分析结果，可以看出6PPD-Q在实际尿液中未出峰，而加标尿样峰形对称，响应良好，说明安装隔离柱后可以严格控制空白。

### 2.3 萃取溶剂的选择

按照1.3节所述前处理方法，替换不同萃取溶剂，对分别加入0.1 ng 6PPD-Q标准品的尿液及灰尘样本进行处理，比较DCM-HEX（1∶1，v/v）、EA、ACN及HEX 4种溶剂对6PPD-Q的萃取效果，每种溶剂平行处理3个样本，处理后样本分析结果如图5所示。4种溶剂萃取对尿样中6PPD-Q回收率排序为EA>DCM-HEX（1∶1，v/v）>ACN>HEX，其中EA对尿样中6PPD-Q的回收率最高，达到94.4%；4种溶剂萃取对灰尘中6PPD-Q回收率排序为HEX>ACN>DCM-HEX（1∶1，v/v）>EA，其中HEX对灰尘中6PPD-Q的回收率最高，达到93.8%。4种溶剂对6PPD-Q回收率排序的差异可能是因为尿样和灰尘样品分别是液态和固态，样品的pH值不同，6PPD-Q存在的状态不同。对于尿液中6PPD-Q的提取，是将其从水相转移至有机相，涉及极性、氢键等作用下的两相分配；而对于灰尘中6PPD-Q的提取，是将其从固体表面吸附的状态洗脱至有机相中。以上均是导致不同样品中6PPD-Q最佳萃取溶剂不同的因素。综合考虑溶剂氮吹效率、环保性等，分别选择乙酸乙酯和正己烷为尿样和灰尘的最佳萃取溶剂。

**图5:**
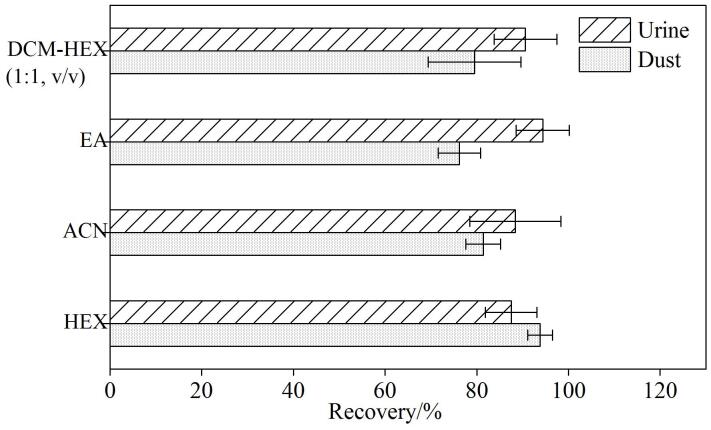
使用不同萃取溶剂时6PPD-Q的加标回收率（*n*=3） DCM： dichloromethane； HEX： *n*-hexane； EA： ethyl acetate； ACN： acetonitrile.

### 2.4 提取次数的选择

在优化的色谱条件下，分别选择乙酸乙酯和正己烷为尿液和灰尘样本的萃取溶剂，按照1.3节的步骤对尿液和灰尘的加标样本进行提取。考察提取2次、3次对于萃取效率的影响。结果发现，尿液加标样本提取2次、3次的萃取效率分别为91.8%和92.6%；灰尘加标样本提取2次、3次的萃取效率分别为92.4%和93.1%。说明提取2次或3次对于6PPD-Q萃取效率的影响不大，从环保、简便的角度考虑，选择提取2次进行后续实验。

### 2.5 基质效应

样品基质中的干扰物会导致基质增强或者基质抑制效应。本实验通过计算空白基质标准曲线与溶剂标准曲线的斜率之比来评价6PPD-Q的基质效应（ME）。当ME为0.8~1.2时，基质效应弱；当ME为0.5~0.8或1.2~1.5时，基质效应中等；当ME<0.5或ME>1.5时，基质效应强。实验结果表明，在使用外标法定量时，尿液和灰尘中6PPD-Q的ME分别为0.282和0.212，均<0.5，有强基质抑制效应。在使用同位素内标进行校正后，尿液和灰尘中6PPD-Q的ME分别为0.947和0.913，均在0.8~1.2范围内，基质效应弱，说明同位素内标校正可以显著改善基质效应的影响。因此，采用内标法对6PPD-Q进行定量。

### 2.6 线性范围、检出限和定量限

配制系列标准溶液，在已建立的分析条件下进行测定。以待测物峰面积与相应同位素内标峰面积的比值（*y*）对质量浓度（*x*，μg/L）绘制标准曲线，以离子比和保留时间定性，内标法定量。所得尿液与灰尘中6PPD-Q的回归方程分别为*y*=2.154 56*x*+0.004 99（*r*=0.999 9）和*y*=0.172 982*x*+0.005 7（*r*=0.999 3），线性关系良好。以3倍信噪比（*S/N*）计算，考虑样本前处理过程，尿液与灰尘中6PPD-Q的检出限分别为0.6 ng/L和0.018 ng/g；以10倍信噪比计算，定量限分别为0.002 μg/L和0.06 ng/g。

### 2.7 准确度和精密度

向空白尿液样品（已加入0.1 ng ^13^C_6_-6PPD-Q）中分别加入0.02、0.10、0.40 μg/L 3个水平的6PPD-Q标准溶液；向空白灰尘样品（已加入1.0 ng ^13^C_6_-6PPD-Q）中分别加入0.50、5.00、100 ng/g 3个水平的6PPD-Q标准溶液。采用优化的条件前处理后进行UPLC-MS/MS上机测试，每个加标样品在日内平行测定3次（*n*=3），计算平均回收率及日内精密度；每个加标样品在第１、2、3 d分别进行测定，计算日间精密度。

结果如表3，尿液和灰尘中6PPD-Q的回收率分别为90.5%~93.0%和90.3%~94.1%，日内精密度分别为0.9%~2.3%和1.8%~5.9%，日间精密度分别为1.1%~5.5%和1.4%~6.3%。本方法具有良好的准确度和精密度。

**表 3:** 6PPD-Q在低、中、高3个水平下的加标回收率、日内精密度及日间精密度（*n=*3）

Matrix	Low			Medium			High		
	Recovery/%	Intra-day RSD/%	Inter-day RSD/%	Recovery/%	Intra-day RSD/%	Inter-day RSD/%	Recovery/%	Intra-day RSD/%	Inter-day RSD/%
Urine	90.5	2.3	4.1	93.0	1.5	5.5	91.2	0.9	1.1
Dust	90.3	5.9	6.3	94.1	3.2	1.4	93.6	1.8	3.5

### 2.8 实际样品的测定

本研究采用已建立的方法对广州市某区120份健康人群的尿液样品中的6PPD-Q进行测定，检出率为74.2%，检出范围为<LOD~13 ng/L，平均值2 ng/L，中位值1 ng/L。目前有关人体尿液中6PPD-Q含量的研究报道非常有限。Du等^［20］^在中国华南地区人体尿液中首次检出6PPD和6PPD-Q，发现6PPD-Q在采集的孕妇（中位值2.91 μg/L）、成人（中位值0.40 μg/L）、儿童尿样（中位值0.076 μg/L）中均有检出。此外，Jiang的团队^［23］^对天津地区成人配对的血浆样本和尿液样本中的6PPD和6PPD-Q进行了检测，尿液中6PPD-Q范围为<LOQ~1.061 μg/g（中位含量0.116 μg/g）。张婧等^［9］^对人体尿液中5种对苯二胺-醌（PPD-Qs）进行了检测，22份尿液中6PPD-Q范围在0.286~0.676 μg/L（中位值0.440 μg/L）。Wu等^［28］^对人体尿液和母乳样本中对苯二胺类污染物（PPDs）及其降解产物PPD-Qs的出现情况进行了调查，发现成人（不包括汽车维修工人）、幼儿园儿童和孕妇尿液中6PPD-Q的平均值分别为1.0、0.21和1.6 μg/L。最近在中国浙江进行的一项研究^［29］^中观察到759名成人尿液中6PPD-Q的平均值为2.76 μg/L，检出范围为<LOD~20.85 μg/L。Dai等^［30］^检测了电子垃圾拆解区2~7岁儿童尿液中6PPD和6PPD-Q的浓度。结果发现，电子垃圾区儿童尿液中6PPD-Q（中位值2.34 μg/L）显著高于参照区儿童（中位值0.24 μg/L）。在已知相关的文献中对尿液样本的处理均未进行酶解，这可能是由于6PPD-Q未在人体内形成结合态代谢物而直接经尿液排出。对于尿液中6PPD-Q的提取，除了Qu等^［29］^的研究采用的是固相萃取法，其他研究均采用的是液液萃取法，本研究采用的是操作更为简便、快捷的液液萃取法。如表4所示，本文建立的方法LOQ较现有方法明显更低，在相同取样量的情况下，本方法对样本的浓缩倍数更高，能够实现尿液样本中6PPD-Q的超灵敏检测。相较已报道的文献，本研究中所涉及的120份尿样中6PPD-Q的检出浓度较低，这可能是由于本研究中的尿液样本来源人群可能未受到6PPD暴露或暴露量较低。

**表 4:** 与其他方法的比较

Matrix	Sample volume/weight	Redissolved volume	Enrichment factor	Injection volume/μL	LOQ	Detected range	Median	Ref.
Human urine （*n*=22）	0.2 mL	50 μL	4	2	0.008 μg/L	0.286-0.676 μg/L	0.440 μg/L	［^9^］
Human urine （adults， *n*=50）	1 mL	2 mL	0.5	5	0.021 μg/L	0.055-2.11 μg/L	0.40 μg/L	［^20^］
Human urine （*n*=100）	1 mL	1 mL	1	5	0.05 μg/L	<LOQ-1.061 μg/g	0.116 μg/g	［^23^］
Human urine （*n*=120）	1 mL	200 μL	5	3	0.002 μg/L	<LOD-13 ng/L	1 ng/L	this study
Indoor dust （*n*=97）	100 mg	50 μL	-	-	0.27 ng/g^a^	0.33-82 ng/g	9.5 ng/g	［^10^］
House dust （*n*=18）	100 mg	-	-	-	0.11 ng/g	<LOQ-0.4 ng/g	<LOQ	［^13^］
Road dust （*n*=22）	100 mg	500 μL	-	-	0.18 μg/L^b^	116-1238 ng/g	809 ng/g	［^14^］
Indoor dust （*n*=31）	50 mg	500 μL	-	3	0.06 ng/g	1.8-24.9 ng/g	3.05 ng/g	this study

a. LOD of methed； b. LOQ of instrument.

采用已建立的方法对广州市内31份室内灰尘样品中的6PPD-Q进行测定，检出率为100%，检出范围为1.8~24.9 ng/g，平均含量为5.23 ng/g，中位含量为3.05 ng/g。如表4所示，与目前报道的文献检出范围相符。与现有方法对比，本文建立的方法具有操作简单、分析时间短和重复性好的优点。同时，表4表明，文献［^10^］的0.27 ng/g为方法检出限，按照该文献LOD是3倍*S/N*计算得到的，则对应的LOQ（*S/N*=10）应为0.9 ng/g，本方法LOQ为0.06 ng/g，较其更低，更有优势；文献［^14^］的0.18 μg/L为仪器检出限，我们的方法仪器检出限可低至0.01 μg/L，较其更有优势。因此，本方法具有更低的检出限，且已成功应用于实际人体尿液和灰尘样品的检测。

综上，本方法适用于人体尿液及室内灰尘中6PPD-Q的测定，操作简便，结果准确。

## 3 结论

本研究建立了人体尿液及灰尘中6PPD-Q的超高效液相色谱-串联质谱同时测定的方法。人体尿液及灰尘样本基质复杂，且尿液中6PPD-Q水平低，6PPD-Q内标的使用，能够一定程度上降低基质效应的影响，减少操作过程引入的误差，结合萃取溶剂的筛选优化，提高了方法的准确度。该方法样品前处理简单、快速，准确可靠，有望为后续人群6PPD-Q的内外暴露水平监测提供技术支撑。
